# Multi-View Hand-Hygiene Recognition for Food Safety

**DOI:** 10.3390/jimaging6110120

**Published:** 2020-11-07

**Authors:** Chengzhang Zhong, Amy R. Reibman, Hansel A. Mina, Amanda J. Deering

**Affiliations:** 1 School of Electrical and Computer Engineering, Purdue University, West Lafayette, IN 47907, USA; reibman@purdue.edu; 2 Department of Food Science, Purdue University, West Lafayette, IN 47907, USA; hminacor@purdue.edu (H.A.M.); adeering@purdue.edu (A.J.D.)

**Keywords:** egocentric video, activity recognition, deep learning, temporal segmentation

## Abstract

A majority of foodborne illnesses result from inappropriate food handling practices. One proven practice to reduce pathogens is to perform effective hand-hygiene before all stages of food handling. In this paper, we design a multi-camera system that uses video analytics to recognize hand-hygiene actions, with the goal of improving hand-hygiene effectiveness. Our proposed two-stage system processes untrimmed video from both egocentric and third-person cameras. In the first stage, a low-cost coarse classifier efficiently localizes the hand-hygiene period; in the second stage, more complex refinement classifiers recognize seven specific actions within the hand-hygiene period. We demonstrate that our two-stage system has significantly lower computational requirements without a loss of recognition accuracy. Specifically, the computationally complex refinement classifiers process less than 68% of the untrimmed videos, and we anticipate further computational gains in videos that contain a larger fraction of non-hygiene actions. Our results demonstrate that a carefully designed video action recognition system can play an important role in improving hand hygiene for food safety.

## 1. Introduction

In this paper, we use multi-camera video analytic methods to design a system that recognizes hand-hygiene actions for food safety. Food safety is a discipline that describes scientific methods to prevent contamination and foodborne illness at different stages of food production. The stages include, but are not limited to, food handling, food storage, equipment cleaning, and staff hygiene. In recent years, where the burden of foodborne illnesses is increasing, evidence indicates that the majority of food contamination is caused by inappropriate food manufacturing practices, involving workers with poor food handling skills [1]. Therefore, we consider video monitoring combined with video-analytic methods for food handling evaluation to be a fast and cost-efficient way to do self-assessment for food growers, processors, and/or handlers.

**Figure 1:**
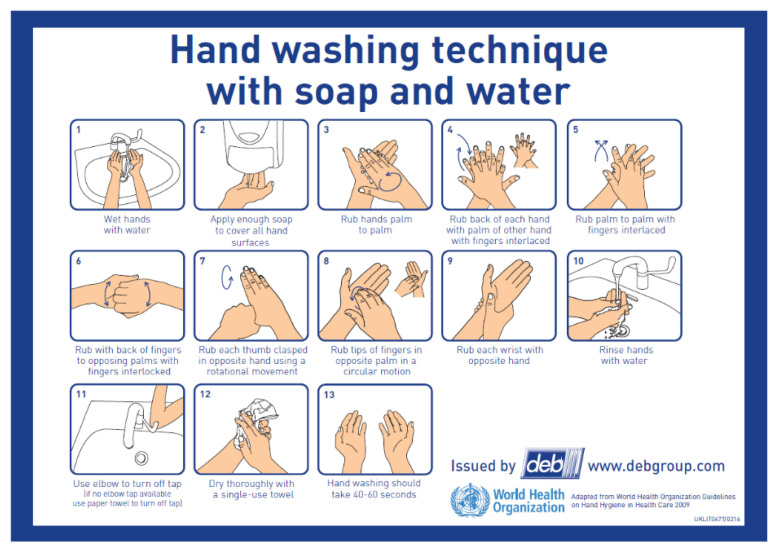
Standard hand-hygiene steps.

In food handling, there are many steps to achieve good manufacturing practices (GMPs). Hand-hygiene is one of the most critical steps. Effective hand-hygiene can reduce food contamination by human pathogens, since this step reduces the likelihood that food handlers harbor pathogenic microorganisms on their hands and transfer them to food products [2]. According to the World Health Organization, there are 12 steps [3] a person should follow to perform effective hand-hygiene. As illustrated in Figure 1, the basic steps include: rinse hands, apply soap, rub hands with a variety of different motions, and dry hands. Our goal here was to use cameras to monitor hand-hygiene activities, to automatically identify both positive activities (like those in the figure) and mistakes that either prevent complete decontamination or lead to re-contamination. These mistakes include not rubbing the hands for the required amount of time, touching the faucet with the hands after washing, and not drying the hands.

There has recently been significant progress in automated methods for analyzing video content, a process called video analytics. Stationary cameras placed in a so-called third-person perspective have been used for surveillance, person and vehicle detection and re-identification, activity recognition, and anomaly detection. When recognizing activities of a person, third-person cameras have the advantage of viewing actions from the side view. First-person, or egocentric cameras are mounted on the person performing the activity, often on their head or chest [4]. These cameras have the advantage of viewing the person’s hands and any objects being manipulated, and are particularly useful to observe subtle hand motions and small objects. However, because they are mounted on a person, these cameras often move chaotically as the person moves. As a result, they may not capture the desired activities, and video processing methods like background subtraction and camera calibration become more difficult [4].

Recognizing activities in videos is a rapidly growing field with wide-ranging applications. Activity recognition techniques have been developed for both trimmed, and untrimmed videos. For trimmed videos, the task is to identify which isolated activity happens in a video clip that has been trimmed to contain only a single activity. For untrimmed videos, the task is not only to recognize the target action, but also to localize it temporally within the clip that may contain unrelated background actions. This process is termed temporal action localization and is often addressed by identifying temporal action proposals [5,6,7]. In this paper, we are interested in processing untrimmed videos.

**Figure 2:**
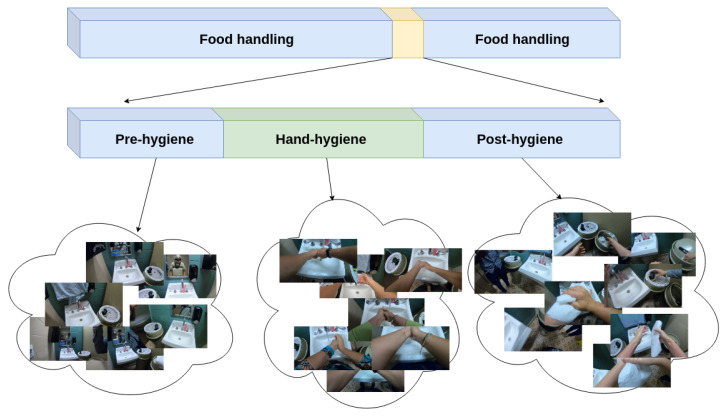
Hand-hygiene periods in untrimmed video.

Figure 2 demonstrates the time-line of hand hygiene within an untrimmed clip that observes the overall process of food handling. Within a period of food handling, which typically lasts at least an hour, hand-hygiene can happen at any time. The yellow period in the figure illustrates when the participant is near the handwashing sink, an obvious requirement for doing hand-hygiene. However, even when they are near the sink, they may not yet be doing hand-hygiene actions. Therefore, we further subdivide the yellow period into three periods as shown in the middle of Figure 2. In the first *pre-hygiene* period, before the participant engages in hand-hygiene, they are near the sink but may be doing any non-hygiene activities. During the second *hand-hygiene* period, the participant stands in front of the sink and performs hand-hygiene actions such as rubbing hands and applying soap. During this period, the participant is likely to stand relatively still and perform one hand-hygiene action after another. Finally, in the third *post-hygiene* period, the participant is likely to move away from the sink and look for a paper towel to dry their hands. Thus, actions occurring in this final period may be a combination of non-hygiene actions and “dry hands”.

Our goal was to design an automated video system that processes untrimmed video to assess the effectiveness of hand-hygiene activities. From the observations of Figure 2, we can recognize that such a system must operate on untrimmed video and quickly identify the narrow time period that actually contains hand-hygiene actions. Therefore, our two overarching design principles are first, to use the most informative camera location to identify each action, and second, to reduce the computational expense without sacrificing accuracy.

In this paper, we propose a framework to recognize hand-hygiene actions inside untrimmed video sequences with the following contributions:

1. We define different levels of tasks for hand-hygiene action recognition, according to their detection difficulty. In this paper, we focus on recognizing standard-level hand-hygiene actions.
2. We record our own hand-hygiene action data set with 100 participants using three camera views.
3. We compare and evaluate the performance of deep-learning models on three different camera views to recognize trimmed hand-hygiene action video clips. We select the best two camera views for our final system design.
4. We empirically explore the required complexity of a CNN model when applied to recognize our selected types of hand-hygiene actions. These empirical evaluation results enable us to deploy appropriate models for our final system.
5. We propose a two-stage framework to recognize hand-hygiene actions from untrimmed video sequences. We combine two camera views to localize and recognize hand-hygiene actions. Taking advantage of the static third-person view camera, we use a low-complexity CNN model to localize the hand-hygiene period within an untrimmed video. Then we apply more complex CNN models to recognize the actual hand-hygiene action types within these candidate locations.
6. Comparing our two-stage system and a baseline system on untrimmed video sequences, we demonstrate that with the support of low-complexity CNN models, our two-stage system achieves nearly equivalent performance by only processing 67.8% of the frames in the untrimmed video. In contrast, the baseline system must process 100% of the frames.

These contributions are relative to our previous work [8], the state-of-the-art in hand-hygiene recognition. There, we introduced the problem of recognizing hand-hygiene actions in egocentric video and introduced one method to address this problem using a single chest-mounted camera. This current work extends our previous work [8] in three main ways. First, we extend hand-hygiene action recognition from solely using egocentric video into a combination of using both egocentric and third-person video. This extension gives us the ability to use a third-person video to effectively localize hand-hygiene temporal regions in untrimmed video, and to recognize each hand-hygiene action with its “expert” camera view. Second, we characterize untrimmed hand-hygiene videos into three temporal periods: pre-hygiene, hand-hygiene, and post-hygiene. Each period includes specific action types, where the majority hand-hygiene actions happen in the hand-hygiene period. To efficiently localize the hand-hygiene period from untrimmed video, we propose to apply a CNN model with a simple architecture to quickly process the untrimmed video to produce candidate temporal regions. Then, we apply a more complex CNN model that only processes these candidates. Third, we introduce our new assessment method toward hand-hygiene action recognition. Due to the special nature of our task, it is not necessary to estimate the duration of all the activities. Instead, for some hand-hygiene action types, we just need to verify that they happened. Thus, in our final result, we evaluate the performance of our system with two different standards.

In Section 2, we introduce previous work on activity recognition from third-person videos and from egocentric videos. In Section 3, we introduce our hand-hygiene data set with three camera views, and describe how it is collected and labeled. We also define the different levels of tasks for hand-hygiene activity recognition. In Section 4, we introduce the overview of our proposed two-stage system. In Section 5, we describe our experiment on evaluating deep learning model performance on trimmed action clips. In Section 6, we evaluate the performance of the entire system. In Section 7, we conclude our work with a brief discussion and summary.

## 2. Related Work

In this paper, our goal is to identify the hand-hygiene steps a user performs within an untrimmed input video. Due to the nature of hand-hygiene activities, highly salient aspects that distinguish hand-hygiene from non-hygiene actions are the person’s hand and arm movements. In addition, different types of hand-hygiene actions have distinctly different motion strength and body parts involved, and these aspects provide important clues to distinguish actions. Therefore, in this section we provide an overview of prior work in activity recognition using computer vision.

Activity recognition focuses on detecting the events inside a video clip and categorizing each into its activity category [9]. Traditionally, researchers were interested in using a detector to identify key points among video clips and construct salient information around these locations [10,11]. One of the most representative methods that follows this methodology is the Improved Dense Trajectory (IDT) [12], which created track points based on dense optical flow across time, and extracted HOG, HOF and MBH features around these salient points. In order to describe complex action types which might have various durations across time, they trained a codebook combined with a linear SVM classifier to make the final prediction. The algorithm achieved the state-of-art in many third-person data sets.

However, in recent years, methods based on deep learning have largely replaced these traditional methods. In general, because traditional hand-crafted feature extraction algorithms are designed by human experts, it is difficult to customize them to different applications. In contrast, deep learning methods can overcome this limitation by using sufficient training data to learn “customized” features. Convolutional neural network models (CNNs) such as AlexNet [13], VGGNet [14], and ResNet [15] apply single 2D images for image classification tasks. Other structures such as long short-term memory (LSTM) [16], two-stream networks [17], two-layer discriminative model [18], CNN-BLSTM network embedded into HMM [19], and PI-based RNN [20] extend the input from spatial information to a combination of spatial and temporal information, which extends the application field of CNN models to video classification. 3D convolutional neural networks have also been applied for action recognition, including C3D [21], I3D [22], T3D [23], and P3D [24]. For long untrimmed videos that contain multi-type actions, the multi-stage temporal convolutional network (MS-TCN) [25] is able to segment different actions temporally.

However, for actions related to hand motions, which are prevalent in hand-hygiene activities, using an LSTM has been shown to be less effective. Köpüklü et al. [26] compare multiple spatiotemporal modeling techniques in combination with 2D CNN in the context of the Jester-V1 [27] and Something-Something-V2 [28] datasets. The result indicates simpler structures such as Multilayer Perceptron (MLP) and Fully Convolutional Network (FCN) outperform methods based on Recurrent Neural Networks (RNNs).

In addition, as portable camera equipment becomes available, researchers have begun to explore action recognition in egocentric videos. Egocentric videos in daily living scenes is explored in [29], using recordings from wearable cameras. Since the daily living scenes considered contain many hand/object interactions, the author proposes to learn an object model that takes into account whether or not the object is being interacted with.

Researchers continue to explore what key information is needed to recognize egocentric activities. One contribution [30] analyzes the important cues to recognize egocentric actions. They experiment with object, motion, and egocentric features and conclude that object cues are crucial clues to classify egocentric actions. Following the idea of recognizing egocentric objects, Ma et al. [31] create an end-to-end CNN model which embeds hand and object information into one system. The model automatically segments the hand regions and localizes objects close to the hands. By merging these data with motion cues, the model achieves good performance. Moreover, hand poses and motion information is also considered in [32]. A compact EgoConvnet is constructed and fused with a two-stream network. However, most of the methods have been tested using datasets such as Georgia Tech Egocentric Activity (GTEA) [33] and Activities of Daily Living (ADL) [29]. These data sets contain clear object cues. For instance, detecting a dish object close to the hand region reveals a salient clue that the action is dish-washing or eating. As mentioned above, the majority of the egocentric action recognition efforts have been constrained within a “hand-to-object” framework, and it is difficult to extend these methods into other egocentric video tasks.

Moreover, in addition to the exploration of actions that involve hand-to-object interaction, actions that only involve pure hand gestures have also been studied. An important approach in this field is to use multi-modalities instead of solely RGB image as the input to the system. One of the crucial modalities is the skeleton joints, which offer a neat and accurate representation of human pose. A multi-model system was designed in [34] for gesture detection and recognition in the 2014 ChaLearn Looking at People dataset [35]. The goal is to recognize 20 Italian sign languages in a video sequence that is captured using a Kinect camera in third-person view. In their system, one of key steps is to do temporal segmentation by identifying the start and end frame of each gesture candidate. Taking advantage of the Kinect sensor, they used the skeleton joint locations as input features to obtain precise information associate with the hand gesture. Combining with an SVM model, the candidate gesture regions can be localized within an untrimmed video sequence. Another work [36] also illustrated the importance of using skeleton joints in recognizing hand gestures. The work is also targeting at the ChaLearn 2014 dataset with multi-model architecture. One of their modalities is to construct a pose descriptor from the skeleton joint locations to describe the global appearance. Recently, Pigou et al. [37] compared the performance of multiple architectures on gesture recognition datasets; their conclusion is that temporal convolution using a recurrent network achieves the best performance. In addition, their results indicate that depth and skeleton information can help improve the detection accuracy in general. Wu et al. [38] propose a Deep Dynamic Neural Networks (DDNN) that processes skeleton, depth, and RGB images as multi-modal inputs. The structure achieves better performance compared to those that only process a single input. Granger et al. [39] compared hybrid NN-HMM and RNN models for gesture recognition when the system input is only the body pose defined by the skeletal joints.

From the studies above, the importance of skeleton joints in recognizing pure hand gestures without the presence of objects is clear. However, skeleton joints extractions are not always feasible. From a camera perspective, all the videos recorded with a special camera like Kinect can naturally provide human skeleton information. While this type of camera has been used in research, it is still not widely used in practical video monitoring. An alternative approach for gathering skeleton data is to through video processing algorithms. OpenPose [40] and DeepLabCut [41] demonstrate the possibility of detecting skeleton joints on humans and animals. However, for our hand-hygiene videos, the third-person camera view normally records the user in close range, so the user’s head and lower body parts are barely visible. Pose estimation methods do not currently robustly detect joints from images containing only the human torso.

Based on the above works, most of the egocentric activity recognition tasks focus on the same category of daily living videos, which involves many hand-to-object interactions. For those works that target pure hand-gesture recognition, the videos are recorded using third-person views with additional depth and skeleton joint information provided. In this paper, we examine how different camera views influence hand-hygiene action detection and design a method that incorporates information from both cameras for hand action recognition in untrimmed video sequences.

Hand-hygiene specifically has been considered by [42,43]. However, hand-hygiene is a small part of their overall plan to support a hospital, which includes pedestrian detection and pedestrian tracking across different cameras. Therefore, hand-hygiene detection is a secondary task in these papers, and their solution is limited to only detecting whether the action happens or not. In this work, we consider a broader set of tasks for hand-hygiene recognition, which requires a new dataset and solution procedure.

## 3. Hand-Hygiene Dataset

In this section, we introduce our hand-hygiene dataset for hand-hygiene action recognition. We start by introducing the video collection procedure. Then, we define the set of actions we consider in this paper, by describing the choice of our specific task. Finally, we describe how a hand-hygiene system should be evaluated in the context of a real application.

### 3.1. Data Collection

As the area of egocentric video becomes popular, researchers have published datasets [29,33] for evaluating the performance of different egocentric action recognition methods. Many publicly available datasets involve only a few participants with recordings done inside home apartments. However, there exists significant differences between a home kitchen and an industrial food handling facility. Moreover, every participant has their own style of hand washing. We believe our data will generalize better if we involve more participants.

To ensure that our dataset includes enough variation between samples, we invited 100 participants and recorded the videos in two separate public restrooms with similar environments (All data collection took place in August 2018 and was done within the context of Purdue IRB # 1804020457.). All participants were allowed to wear any type of clothing, including watches and hand jewelry, and had varied ages, genders, and races. Each participant was recorded twice while they washed their hands. Initially, each participant performed a naive hand washing in the first room, according to their typical hand washing style. Then, the participant was asked to read the instructions for hand-hygiene shown in Figure 1. Finally, each participant was recorded washing their hands again in the second room. When the data were collected, all participants indicated their willingness to have their data published. However, not all participants agreed to a public dissemination of their data; therefore, our hand-hygiene dataset will not be made publicly available at this time.

Our overall goal was to design a method that will operate in any indoor environment, including temporary environments with portable wash-stations. A single camera mounted on the ceiling created a top-down view for the previous work in [42,43]. However, the layout may not be consistent across all indoor environments; for example, the location of faucet, the height of the ceiling, the location (or existence) of a mirror, may all be different for different environments. Therefore, instead of designing a particular camera installation plan for every potential environment, we choose to collect our data in “portable” way. Thus, we use cameras that can be easily mounted on a participant or easily moved from one location to another.

Two type of cameras, egocentric and third-person, are applied in our data collection. An egocentric camera is capable of capturing subtle motions of hands and fingers, which provides supportive information to classify different types of hand actions. In contrast, a third-person camera is efficient at capturing a participant’s body motion as well as any interaction with the surroundings.

To explore the efficiency of third-person video and egocentric video in hand-hygiene actions, we used both egocentric cameras and a static third-person view camera for video recording. Each participant wore a GoPro camera with a harness on their chest as one egocentric camera and an IVUE glasses camera on their nose as another egocentric camera. The third-person view camera was a GoPro camera placed on top of a flat platform near the sink. We will refer these three camera views as “chest camera view”, “nose camera view”, and “wall camera view” for the rest of this paper. Each video has 1080p resolution, 30 FPS, and a wide viewing angle.

### 3.2. Hand-Hygiene Action Definition

To the best of our knowledge, we are the first project to apply video analytic methods on hand-hygiene actions. Therefore, we want to define the different types of hand-hygiene actions and the purpose of analyzing these actions. We defined three different video analytic tasks to explore in hand-hygiene actions. Based on the level of difficulties of these tasks, we define them as *detail-level*, *standard-level*, and *detection-level* tasks. In this paper, we will only explore the standard-level task.

#### 3.2.1. Detail-Level Hand-Hygiene Task

In the detail-level task, the goal is to strictly follow each of the steps outlined by the World Health Organization in Figure 1; did a participant perform each of the 12 steps?

**Figure 3:**
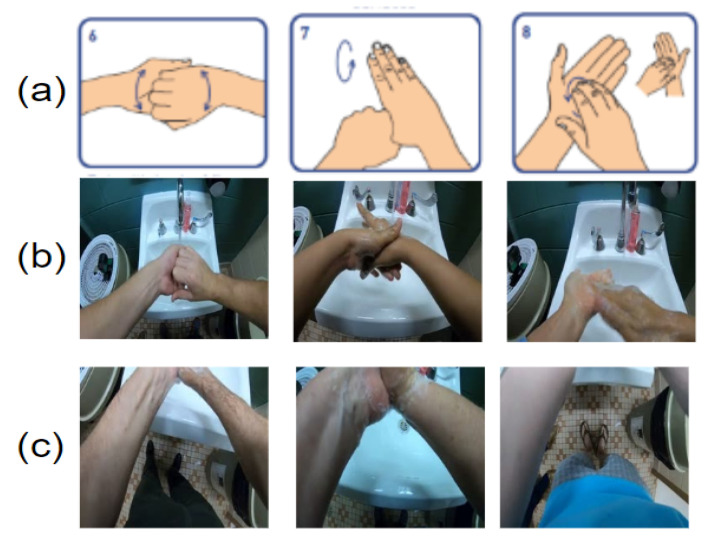
(**a**) Standard subtle actions between fingers. (**b**) Clear view of subtle actions. (**c**) Hands out of camera views.

This detail-level task has the highest difficulty compared to other hand-hygiene tasks, especially for those actions that involve subtle motions of the hand and fingers, such as those illustrated in Figure 3a. As indicated in Figure 3b,c, the egocentric camera does not always capture the entire hand regions, because participants have different body size and personal habits. Therefore, it is inevitable that hand regions may be missing during the detailed hand-hygiene steps.

Even if the entire hand regions are clearly recorded, recognizing actions with subtle finger and hand motions is still difficult. The temporal boundaries between actions of rub cross finger, rub palm, and rub thumb are difficult to distinguish even by a human expert. The method in [44] to recognize dynamic long-term motion is only likely to be able to recognize the entire action sequence. To accurately localize the boundary between these similar actions, we may need to apply an RGB-D sensor and construct hand-finger skeleton models [45].

#### 3.2.2. Standard-Level Hand-Hygiene Task

**Figure 4:**
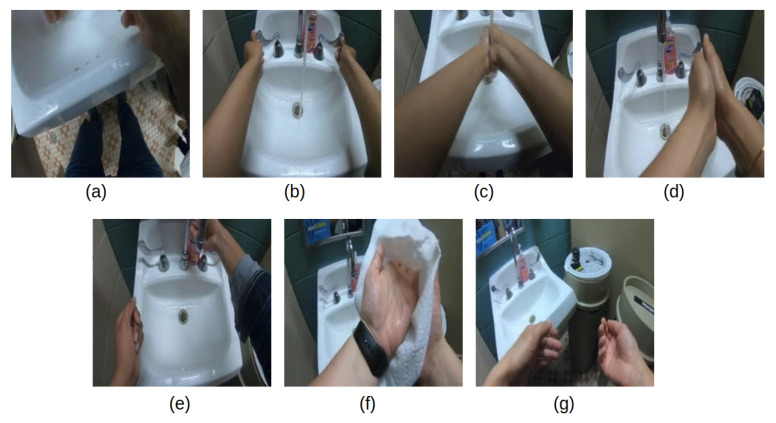
(**a**) Touch faucet with elbow, (**b**) touch faucet with hand, (**c**) rub hands with water, (**d**) rub hands without water, (**e**) apply soap, (**f**) dry hands with towel, (**g**) non-hand-hygiene action.

In the standard-level hand-hygiene task, we focus on analyzing only the components of the 12 steps in Figure 1 that are most critical from a food-safety hand-hygiene perspective. As a result, we define the standard-level hand-hygiene task to distinguish among the 7 types of actions shown in Figure 4: touch faucet with elbow, touch faucet with hand, rub hands with water, rub hands without water, apply soap, dry hands, and a non-hygiene action. Essentially, the six rubbing actions are condensed into a single rubbing action, and we retain the critical components of applying water and soap, rubbing for an extended period, and drying the hands. In addition, this task includes identifying the action “touch faucet with hand”, which must be avoided to prevent re-contamination after the hands have been rinsed with water [3].

As mentioned above in Section 3.2.1, the subtle hand and finger motions may not be completely recorded for a variety of reasons. The standard-level task removes the need to distinguish among the subtle hand and finger motions, so a classifier for these 7 action classes will be more robust than for the detail-level task, both with respect to the variations of a participant’s body size and to the hands not appearing in the camera view.

#### 3.2.3. Detection-Level Hand-Hygiene Task

In addition to the previous two task levels we defined, there exists a detection-level hand-hygiene task, which simply analyzes whether or not the hand-hygiene action happened. For this task, egocentric camera information regarding the hands is not necessary, and directly analyzing the third-person camera should achieve an acceptable result.

### 3.3. Evaluating Hand Hygiene for a Real Application

In this paper, we focus on the standard-level hand-hygiene task, which considers 7 different action types. In a real application for hand-hygiene verification, a user would like to know if some actions happened for a sufficiently long time, and if other actions happened at all. Therefore, performance evaluation of a system requires distinct measurements depending on the action type. For example, both “rub hands with water” and “rub hands without water” reflect the participants’ effort to clean their hands. Thus, it is important for us not only to detect the existence of these actions, but also to determine how long they last. Three actions, “touch faucet with elbow”, “apply soap”, and “dry hands”, help sanitize and prevent re-contamination; therefore, we only need to confirm the existence of these actions. However, “touch faucet with hands” actually re-contaminates hands, and should be identified if it happens. Meanwhile, the background non-hand-hygiene actions do not influence the hand washing quality, and these are included in the set of actions for completeness.

Based on these observations, we evaluate the hand-hygiene performance of a participant by evaluating whether a correct decision was made regarding which action occurs during each second of the video. Thus, we divide each target video into non-overlapping units of 30 consecutive frames, which corresponds to 1 s in time. Each unit is labeled with only one action type by counting the most frequent action type of each frame among all 30 frames. This assumes a detector makes one prediction during each unit. To achieve this, a detector can predict an action for 30 frames individually and average the confidence scores to create a prediction result for the unit, for example.

**Figure 5:**
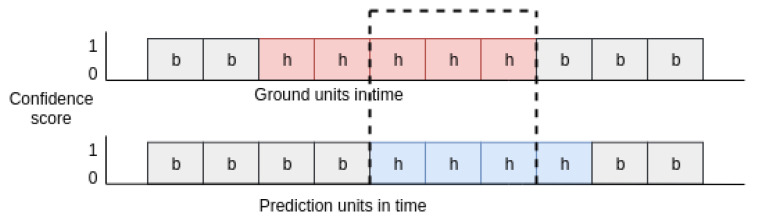
Unit-level prediction. **Top**: ground truth labels. **Bottom**: prediction results. Each rectangle represents consecutive 30 frames. Label “b” is a “non-hygiene” action and label “h” a is “hand-hygiene” action.

The top and bottom of Figure 5 illustrate the unit-based ground truth and prediction results, respectively. The region between the dashed lines is the intersection between ground truth label and system prediction of a hand-hygiene action. If, for this particular action, we only need to assess whether it happened or not, we can simply verify the existence of an intersection region. However, if for this action, we need to assess how long it lasted, we evaluate the prediction result by the Jaccard index, which is also known as the Intersection Over Union (IOU). The Jaccard index is defined as $J=\left(R_{n}\;\cap \;B_{n}\right)/\left(R_{n}\;\cup \;B_{n}\right)$, where $R_{n}$ is the number ground-truth units and $B_{n}$ is the number of predicted units, for a particular action.

## 4. A Two-Stage System to Evaluate Hand Hygiene

In this section, we describe a basic two-stage system to detect standard-level hand-hygiene actions, namely, the 7 actions described in Section 3.2.2, that occur in real-life scenarios, i.e., in untrimmed videos. Our two-stage design is motivated by the desire to apply low-complexity processing during a first pass, with the goal to reduce the amount of video that later must be processed with more complex methods in the second stage. Both stages process each of the wall and chest camera outputs, and the specific tasks of each stage are motivated from our experiments described below in Section 5. In particular, the two stages and selection of which camera should be applied to a certain hand-hygiene stage are motivated by our experiment (described below in Section 5.1) which explores which camera is most effective for each action, and by our observations from Figure 2 above.

Recall that most hand-based actions are densely located in the hand-hygiene period and partially distributed within the post-hygiene period. All the remaining video content consist of non-hygiene actions with unknown and variable duration. Thus, it would be inefficient to densely process the entire untrimmed video with a computationally-complex CNN model. Recall that an untrimmed video clip contains more than one action type, while a trimmed video clip contains only one action.

The first stage of the system consists of two so-called coarse classifiers; one processes the wall video and one processes the chest video. They each densely process the entire untrimmed video and localize potential candidates for the temporal regions that might contain standard-level hand-hygiene actions. In the second system stage, we apply two so-called refinement classifiers that only process the candidate locations identified in the first stage.

**Figure 6:**
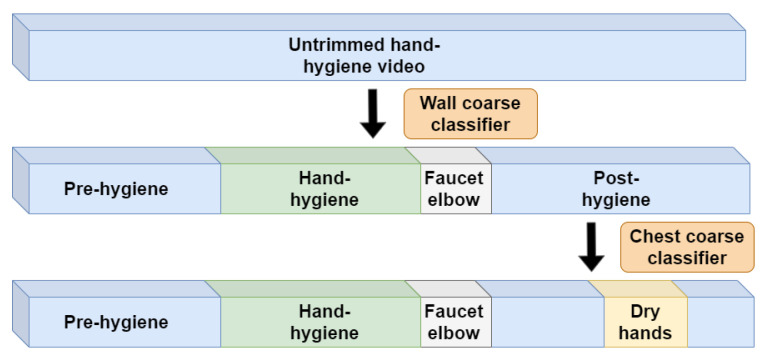
System stage 1: Untrimmed hand-hygiene video processing with coarse classifiers.

Specifically, in the first system stage, as shown in Figure 6, we apply the wall coarse classifier to densely process the entire untrimmed video in non-overlapping 30 frame units. Even if a non-overlapping split might cut a small portion of consecutive hand-hygiene action into two different units, this will have little affect on our goal here, since when our system is applied in practice, whether the hands are detected as rubbing for 10 or 10.1 s will not influence on the final hand-hygiene quality. The entire untrimmed video is then divided into pre-hygiene, hand-hygiene, post-hygiene regions, and candidate regions of the “faucet elbow” action. The pre-hygiene region will not be further processed later in the system, but the other regions will be processed by subsequent specifically-targeted classifiers. Design considerations for the wall coarse classifier are described in Section 5.3.1.

Additionally in the first system stage, the chest coarse classifier processes only the region identified as “post-hygiene”. Its goal is specifically to identify whether the action of “dry hands” happened or not. Further detail on its design is provided in Section 5.3.2.

**Figure 7:**
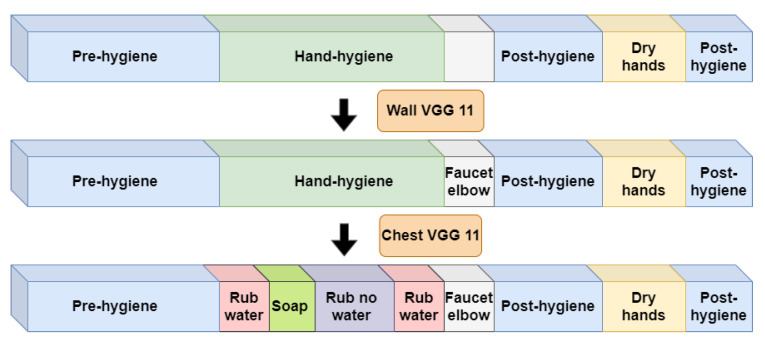
System stage 2: Trimmed hand-hygiene video processing with refinement classifiers.

In the second system stage, shown in Figure 7, we apply two refinement classifiers. The first wall-refinement classifier only processes the short temporal region that was identified by the first-stage wall coarse processor as being a candidate region for the “faucet elbow” action. Its goal is simply to verify the existence of the “faucet elbow” action and further refine its temporal location.

The final classifier uses the chest view to refine the actions that take place in the hand-hygiene region. Its goal is to label every time unit according to each of the 7 actions in the standard-level hand-hygiene task described in Section 3.2.2. The design of this classifier is considered in Section 5 below. In particular, as we show in Section 5.1, the chest camera view provides rich details for hand actions during the hand-hygiene period. Therefore, this classifier is well suited for identifying the actions that the earlier classifiers have not considered, namely, the 4 actions “touch faucet with hand”, “rub hands with water”, “apply soap”, and “rub hands without water”.

**Figure 8:**
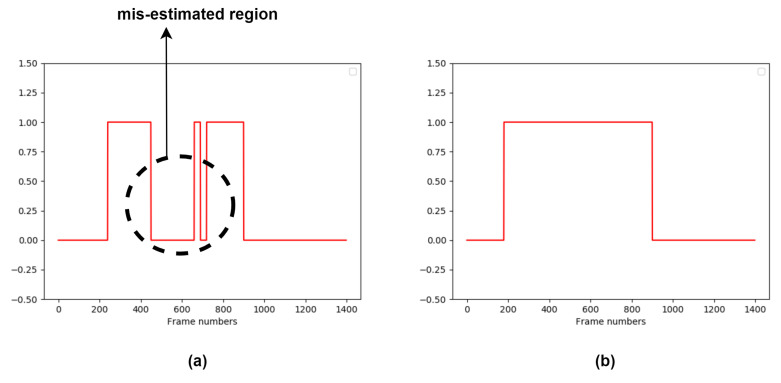
Temporal location of hand-hygiene temporal (**a**) produced by the first system stage (**b**) and final searched region. Each plot shows the confidence score of deciding “hand hygiene” or not as a function of the number of frames. The dashed circle indicates the region that is misidentified by the wall coarse classifier.

However, some actual hand-hygiene periods may have been misidentified as non-hygiene in the first system stage. This is illustrated in Figure 8. To compensate for this possibility, we expand the hand-hygiene temporal region by applying an iterative search method. In particular, we apply the chest refinement classifier to all time units to the left and right of any identified hand-hygiene time unit. This continues recursively, until this classifier labels a time unit as either a non-hygiene action or “touch faucet with elbow”, and all the initially-labeled hand-hygiene units have been processed. An illustration of the final temporal region searched by the chest refinement classifier is indicated in Figure 8b.

To summarize, the overall system takes as input the untrimmed video that contains both hygiene and non-hygiene actions. Four actions must be identified as to whether they happen or not. These are the actions of “dry hands”, “touch faucet with elbow”, “touch faucet with hand”, and “apply soap”. The first is detected by the chest coarse classifier, the second by the refinement wall classifier, and the latter two by the refinement chest classifier. For the remaining two actions, “rub hands without water” and “rub hands with water”, both of which are identified by the refinement wall classifier, it is important to verify that they lasted for at least 20 s. During our final system evaluation in Section 6, we will consider estimates of this duration as a measure of performance. However, in the next section, which explores detailed questions about how to design each of the four classifiers, we consider only detection and recognition accuracy.

## 5. Design and Evaluation of Individual Classifiers in the Two-Stage System

In this section, we explore the design of each individual classifier in the the two-stage system described in the previous section. We take an experimental approach to address the following questions:

- Question 1: Which camera is most informative for which actions? (Section 5.1)
- Question 2: How much computational complexity is required for the standard-level hand-hygiene task? What models should we use? How deep? How much accuracy do we lose if we use classifiers with lower computational complexity? (Section 5.2.1)
- Question 3: Is RGB information sufficient or should we include motion information? (Section 5.2.2 and part of Section 5.3.1)
- Question 4: To coarsely recognize hand-hygiene temporal regions from untrimmed input video, what model should be used for the wall camera? (Section 5.3.1)
- Question 5: To coarsely recognize the single action of “dry hands”, can we use hand-crafted features or would a CNN perform better? (Section 5.3.2)

First in Section 5.1, we compare the performance of using wall camera, nose camera videos, and chest camera videos on solving the standard-level hand-hygiene task which recognizes 7 hand-hygiene actions. After determining that the chest and wall camera are the best two camera views, we explore how sophisticated a model needs to be to solve this task for both camera views in Section 5.2. This design comparison across well-known CNN structures leads us to select the models for both refinement classifiers. Finally in Section 5.3, we design simpler structures for the coarse classifiers for both these camera views.

### 5.1. Camera-View Comparison for Hand Hygiene

**Figure 9:**
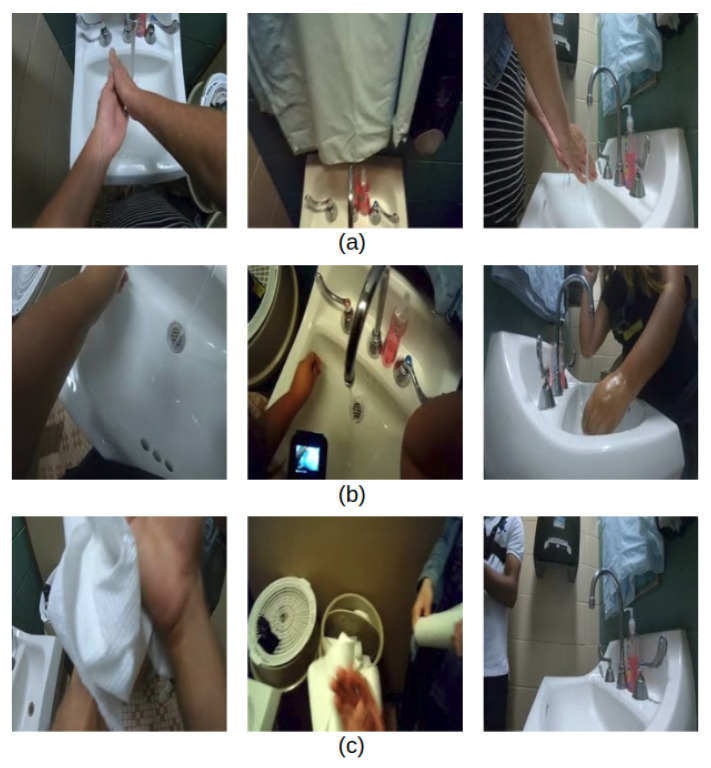
Images from chest camera (left), nose camera (middle), and wall camera (right): (**a**) “rub hands without water”; (**b**) “touch faucet with elbow”; (**c**) “dry hands”.

As indicated in Section 3.1, our dataset consists of video data from a third-person camera and two egocentric cameras. As a result of the camera placement, each camera view has its advantage for recording certain types of actions. As demonstrated in Figure 9b, the chest camera view fails to capture the participant’s arm when the pose involves body motion. In addition, in Figure 9c, the wall camera view can only record half of the participant’s body when the participant walks away from the sink. The nose camera is designed to record the same video as the user’s eye view. However, due to the nature of our hand-hygiene task in a narrow space, the user’s gaze does not always align with the camera view. Thus, some of the nose camera videos do not actually show the hands; an example is shown in Figure 9a.

Our goal in this section is to better understand which camera view is most effective for each action. Therefore, we apply a single model for each camera; the input to each model the set of RGB images from a trimmed video, and the output is one of the 7 actions for the standard-level hand-hygiene task. Since, for hand-hygiene actions, the majority of the content is composed of hand-to-hand and hand-to-arm interactions and there are no salient objects to help distinguish each action type, we choose to use a single deep-learning based model to learn an efficient representation. In this paper, we follow the idea of the two-stream network [17] to consider a 2D CNN structure that processes both RGB image and motion information for activity recognition. In particular, for this exploration, we apply ResNet152 [15] to be consistent with our previous work [8] where we considered only the view from the chest camera.

In the rest of the subsection, we describe our experimental design, the model training, and finally the results of applying the model to our dataset.

#### 5.1.1. Experimental Design

To solve the standard-level task defined in Section 3.2.2, we manually labeled our hand-hygiene video data at a frame level with 7 action types to create ground truth. Since our 100 participants were each recorded twice, this provides us with around 200 videos for each camera view. To verify the system’s robustness against different subjects, the training, validation, and testing data were randomly selected among 100 participants with 66, 12, and 22 people, respectively. This partition and random selection was performed 5 times to create the 5 trials that we will refer to throughout this paper. We further trim each video into clips based on the frame-level ground truth of actions types. As a result, each contains only one action type for its duration.

#### 5.1.2. Model Training on RGB Images

For each camera view, we begin with a ResNet152 model that was pre-trained on ImageNet [46]. We fine-tune the model so its last fully-connected layer outputs one of our 7 action classes. As a result that our data were all recorded in two nearly identical environments and because we are interested in a pure comparison between the different camera views, we do not apply data augmentation techniques like image scaling or random crop for training. However, since the wall camera was placed on one side in the first room and the opposite side in the second room, we horizontally flip its frames to the same direction. This improves both training and testing efficiency.

The training hyperparameters for each camera view are set to the same for comparison, although they could be further optimized. However, our goal for this experiment was to compare the efficiency of each camera when processed by the same CNN architecture. Each model was trained for 250 epochs using a Stochastic Gradient Descent (SGD) optimizer with learning rate 0.001. The learning rate was decreased by a factor or 10 at 100 and 200 epochs. The batch size is 25, and each sample in a batch is a randomly selected video frame from a trimmed training video clip.

#### 5.1.3. Model Evaluation on RGB Images

Testing is performed on each trimmed video clip. The trained model is applied on every frame of a test video clip, followed by a softmax function. The average score among all frames indicates the prediction result. The testing results of all 7 actions averaged over the 5 trials is shown in Table 1, for each of the three camera views.

Comparing across the rows of the table, we can see that the chest view outperforms the other views for recognizing actions with detailed hand-hand interactions, such as “rub hands without water”, “touch faucet with hands”, and “dry hands”. However, we observe a small performance drop for the chest view on the actions “rub hands with water” and “apply soap” compared to the wall view, because the chest camera does not always capture the hand regions.

Compared to the chest camera view, the nose camera view achieves less accurate results for all action types except “apply soap”. This is because the nose camera is mounted above the chest camera, so it is easier for the nose camera view to capture the scene when the user applies soap.

**Figure 10:**
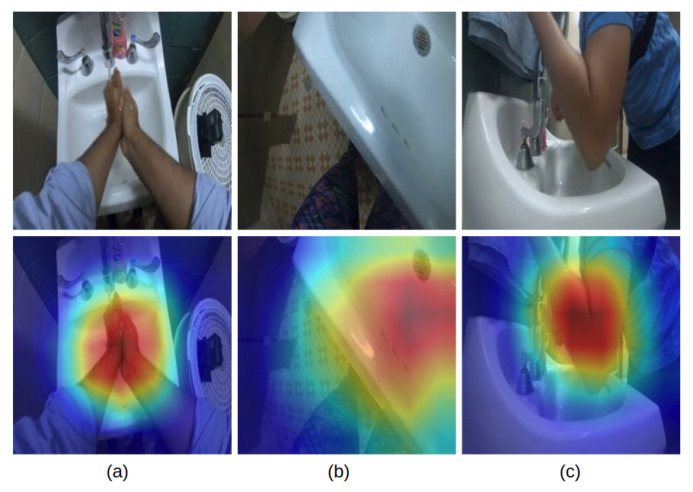
Grad cam [47] results of (**a**) chest cam: rub hand with water, (**b**) chest cam: touch faucet with elbow, (**c**) wall cam: touch faucet with elbow, when applied to the respective model.

On the other hand, the wall camera can also predict many of the action types within its viewing range, especially when an action contains the body motion from a participant. As the shown in the heatmaps in Figure 10a–c, the chest camera can accurately capture human hands. But due to the limitation of the camera angle, the chest camera cannot capture the salient region of the arm as well as the wall camera can. Thus, the CNN model from the chest camera view predicts the “touch faucet with elbow” action by focusing on the sink region. This may be effective for the current action set where only one action contains significant body motion, but in general, the chest camera view will not be robust to body actions. As a result, the wall camera outperforms the chest camera by about 10% on the “touch faucet with elbow” action. Another drawback of the wall camera view is also obvious if we consider the “dry hands” action. For this, a participant is likely to move around the room while they wipe their hands with a paper towel. The failure to track the participant causes the wall camera’s low prediction accuracy of 54% on detecting “dry hands”.

The last row of Table 1 summarizes the average accuracy over all 7 actions for each camera view. Due to the disparity in the number of videos for each action type, the wall camera’s advantage is not reflected by the average accuracy. However, it is undeniable that the wall camera performs best for recognizing actions related to body motion. Overall, to answer Question 1 in the beginning of Section 5, we conclude that the chest camera view is effective at recognizing hand-hand related actions and the wall camera view can be applied to recognize body actions to monitor the presence of a participant within its viewing area.

### 5.2. Model Comparison for Refinement Classifiers

In the last section, we applied the ResNet152 model to explore the relative advantages of using the wall and chest camera views for a standard-level hand-hygiene task. ResNet152 is a relatively complex model; therefore, in this subsection, we evaluate the performance of different CNN models on the standard-level hand-hygiene task. In addition, we also evaluate whether adding optical flow improves performance for the chest camera. Together, these experiments inform the design of the refinement classifiers in the second stage of our system.

#### 5.2.1. Model Comparison for RGB Images

The models we consider are VGG19, VGG16, and VGG11, which are variants of the VGG network [14] with high to low structure complexity. Again, we apply pre-trained models and fine-tune the last fully-connected layer to output 7 action classes. For comparison purposes, the training settings of VGG19, VGG16, and VGG11 are exactly the same as ResNet152 from Section 5.1.2.

The testing results of data from trial 1 (only) are listed in Table 2. The model accuracy is evaluated as the number of true positive and true negative predicted video clips divided by the total number of video clips. As we can see, the overall detection accuracy from both wall camera and chest camera view have minor variations as the model complexity drops, and the final performance of VGG11 is similar to that of ResNet152. This is mainly because the scenario we currently consider is limited to a public bathroom environment with similar camera angles. Therefore, the less complex CNN architecture can still achieve reasonable detection accuracy. This decision may have to be revisited if the model is tested with different camera angles in different environments. However, for the current scenario, these results suggest that the answer for Question 2 in the beginning of Section 5 is that VGG11 with an input RGB image is adequate for the standard-level hand-hygiene task as a refinement classifier.

#### 5.2.2. RGB and Optical Flow Comparison

Based on the result from Section 5.1.2, we have demonstrated that chest camera RGB-image model has an advantage for analyzing the hand-hygiene actions that specifically concern the hands. Many previous works have demonstrated the importance of including motion information for egocentric activity recognition [31,32] and third-person activity recognition [17]. Thus, we conducted an experiment to explore the degree to which incorporating motion information helps to interpret egocentric hand-hygiene videos. For this, we create optical flow images using the TV-L1 optical flow [48] implementation [49].

**Training:** Similar to the chest camera RGB model, the chest camera optical flow model still takes a pre-trained ResNet 152 network and fine-tunes the last fully-connected layer for our 7 action classes. The first convolutional layer is revised to take as input 10 frames of horizontal and vertical optical flow images. The model is trained with 350 epochs and learning rate 0.001. The learning rate was decreased by 10 at 200 and 300 epochs. The remaining hyperparameter settings are the same as above.

**Testing:** The testing step follows the same procedures as Section 5.1.2 on each trimmed video clip, except that the trained model processes every 10 optical flow frame pairs instead of a single RGB frame. The testing results of all 7 actions averaged across the 5 trials are listed in Table 3.

We see from the table that for all 7 actions classes, using optical flow does not provide significant improvements for recognition. In addition, in our previous work [8], fusing the RGB and optical flow models did not show a meaningful boost in recognition accuracy. Therefore, to answer Question 3 in the beginning of Section 5, we conclude that the adding temporal motion information is not necessary for the standard-level hand-hygiene task.

### 5.3. Model Design for Coarse Classifiers

In this section, we develop classifiers to recognize coarse hand-hygiene actions in the first system stage described in Section 4. The actions to be recognized in this stage are easier to distinguish than in the second stage. Thus, we can apply less complex CNN architectures in this stage. The designs for the coarse wall and chest classifiers appear in Section 5.3.1 and Section 5.3.2, respectively.

#### 5.3.1. Wall Camera Coarse Classifier

The wall camera (a third-person view camera) is placed on a flat platform in our experiment, so it captures the participant’s actions near the sink from a close range. Due to the limitation of the camera angle, this camera view cannot capture hand actions in detail; however, we showed in Section 5.1 that it is useful at providing participant’s body actions and location. We believe the wall camera view is suitable for coarsely localizing both the hand-hygiene period and the action of touching the faucet with an elbow. Thus, the goal of the wall coarse classifier is to predict 3 types of action classes: “touch faucet with elbow”, hand-hygiene, and non-hygiene. The non-hygiene actions that happen before the first hand-hygiene action is identified are categorized as pre-hygiene actions, while those identified after are called post-hygiene.

Based on the observations in [32,42], shallow CNN models with 2 convolutional layers and 2 fully connected layers are effective for recognizing hand actions in both egocentric and third-person camera views. However, simply applying an RGB-based model ignores potentially useful information about motion. Indeed, motion information [17] or multi-modality depth and skeleton [36] information has been shown to improve detection accuracy for action recognition. Therefore, we propose to use a simple CNN model for this coarse classifier to quickly process the untrimmed video, and we also explore whether the addition of motion information can improve accuracy. Designs based on these two considerations are described next. The performance of these design choices is then compared to finalize our design of the wall camera coarse classifier.

**Model for RGB images:** To ensure a low computational cost and fast processing speed for this coarse processor, we are inspired by the tiny image dataset CIFAR 10 [50]. Thus, we explore CNN structures that take as input a down-sampled image of size 32 × 32. The basic architecture of the model follows the design of the VGG networks with a 3 × 3 kernel size and max pooling kernel size of 2 × 2 and stride 2.

**Figure 11:**
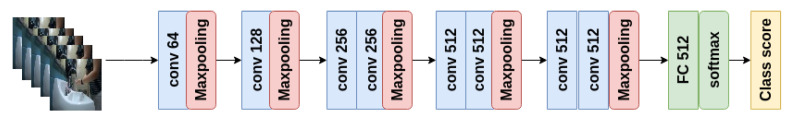
Coarse wall classifier: Model with RGB only.

To explore how variations of the CNN architecture affect the detection performance, we evaluate three structures. The first structure consists of 5 groups of convolutional layer, max pooling layer, and RELU non-linearity followed with a fully connected layer and softmax as indicated in Figure 11. The second structure is modified from the first by changing the last group of convolutional layers with 512 output channels into a fully connected layer, which results in 4 groups of convolution layers followed by 2 fully connected layers. In the last setting, we attempt to further reduce the model complexity by replacing the 4th group of convolutional layers with depthwise separable convolutional layers [51].

**Features to describe supplemental silhouette information:** Since our wall camera is stationary, foreground–background segmentation is a powerful method to understand moving objects in the scene. Three methods we consider here are an adaptive background learning [52] method, the Fuzzy Choquet integral [53] and SuBSENSE [54]. We compute these using the implementation in [55].

In addition, we consider the motion history image (MHI), which is an efficient method to create a temporal template that indicates motion [56]. MHI encodes the motion density from past frames into the current frame. It is a robust representation of human gestures especially under static camera view. This feature has three parameters to set: the motion history duration τ, the decay parameter δ, and a threshold ξ [56]. In our experiment, these are set to τ=5, δ=1, and ξ=20, respectively.

**Figure 12:**
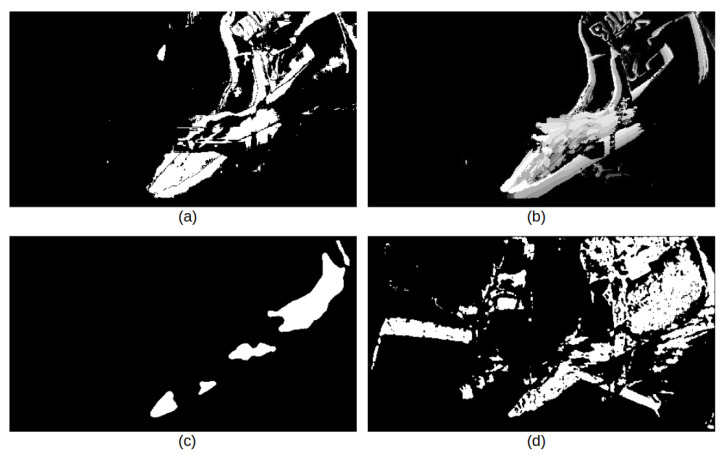
(**a**) Adaptive background learning [52]. (**b**) Motion history image. (**c**) Fuzzy Choquet integral [53]. (**d**) SuBSENSE [54].

Figure 12 shows the three different background detections along with the MHI for one frame. Compared with the fuzzy method and SuBSENSE method in Figure 12, the basic adaptive background learning method and motion history image appear to be more robust for isolating the human silhouette. Overall, the motion history image shows the best results of tracking the human silhouette with less background noise. Therefore, we select motion history image as an additional input for the wall camera coarse classifier.

**Figure 13:**
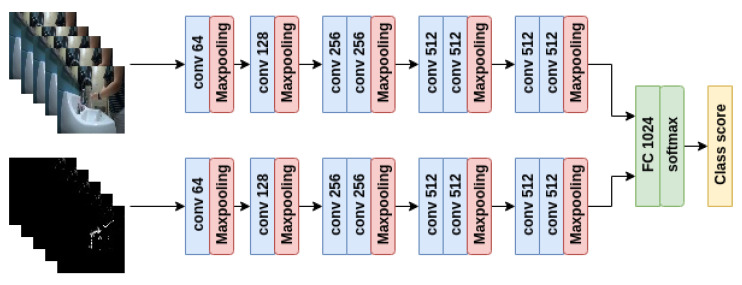
Coarse wall classifier: Model with RGB and motion history image (MHI).

**Model for both RGB images and MHI:** In addition to the three RGB-only models mentioned above, we also consider a model that takes RGB and the motion history image as indicated in Figure 13. MHI is computed using RGB images of size 224 × 224 and down-sampled to 32 × 32 size to be input to the model. We use the same architecture for both the MHI and the RGB image. The output feature maps from the last convolutional layer are concatenated with the feature maps from RGB model and passed into the final fully-connected layer.

**Model training:** All the training and testing are performed on the first data trial defined in Section 5.1.1. We applied the same hyperparameters settings for the model that uses only the RGB as well as the model that uses both RGB and MHI. All models are trained from scratch without using pre-trained weights. For fast convergence and to reduce over-fitting, batch normalization [57] is applied to the output of every convolutional layer before it enters the ReLU non-linearity. Each model is trained by 250 epochs using Stochastic Gradient Descent (SGD) optimizer, cross entropy loss, and learning rate 0.001. The learning rate was decayed by 10 at 100 and 200 epoch. Batch size is selected at 128 and each sample in the batch is a randomly selected video frame from a trimmed training video clip.

**Testing:** To compare the performance of these structures, we consider untrimmed testing videos and predict 3 action types: “faucet elbow”, hand-hygiene, and non-hygiene. We evaluate the performance of each structure with two metrics: the frame-level accuracy and the unit-level accuracy. Frame-level accuracy is computed by the sum of true positive and true negative predictions over all frames in the video. Instead of predicting every single frame, the unit-level prediction starts by cutting the untrimmed video into non-overlapping units of consecutive 30 frames, which is 1 s in our video. Then, we make a prediction of an action class for each unit by averaging each frames prediction confidence score. The unit-level accuracy is also computed as the sum of true positive and true negative units over the total number of units in the video.

The prediction results of the three structures for RGB-only, as well as for the RGB + MHI model are shown in Table 4. As can be seen, among the three different structures that have RGB-only input, the structure with 5 groups of convolutional layers combined with 1 fully connected layer has better performance than others. Notably, the structure with depthwise separable convolution experienced a large performance drop. In addition, the structure with both RGB + MHI achieves better performance than using only the RGB modality. These two architectures are further compared in Table 5, which indicates that incorporating the MHI markedly improves performance for the “faucet elbow” action and the hand-hygiene action. Therefore, to answer Question 4 in the beginning of Section 5, we select the RGB + MHI to be the model for the wall coarse classifier.

#### 5.3.2. Chest-Camera Coarse Classifier

As described in Section 4, after applying the wall coarse classifier on the untrimmed hand-hygiene video, three temporal periods are identified. In the “post-hygiene” period, the participant is expected to dry their hands with a paper towel. However, the wall camera cannot predict accurately whether the “dry hands” action exists or not. Therefore, in this section, we design a system to process the videos from the chest camera, but only in the post-hygiene temporal region to detect the “dry hands” action. Since we only need to confirm if the “dry hands” action happens or not, we anticipate that a low-complexity model is sufficient for this task. A low-complexity model is also desired because the exact location of the “dry hands” action could be anywhere in the long post-hygiene region.

Several options are available for low-complexity models. For temporal segmentation of egocentric videos, Bolaños et al. [58] apply color descriptors to detect the action “in transit”. They computed the color histogram for each video frame and use the difference between histograms as a feature to describe changes in the camera angle. Moreover, Azad et al. [59] argued that hand-crafted features have an advantage over deep-learning methods when applied to small data sets. They achieve good performance by using Histogram of Oriented Gradients (HOG) [60] and Local Binary Pattern (LBP) [61] descriptors on a depth motion map to recognize hand gestures. Therefore, to recognize the “dry hand” action within the post-hygiene period, we propose to test both deep-learning features and the hand-crafted features of color histogram, HOG, and LBP.

**Training:** Similar to the experiments in the previous section, all training and testing are performed on the first data trial defined in Section 5.1.1. For the deep-learning model, we apply the same architectural structure of five groups of convolutional layers with a fully connected layer chosen for the wall coarse classifier in Section 5.3.1. The hyperparameters and training steps are also the same as mentioned previously. Since the model is designed to predict only “dry hand” and “non-hygiene” actions, we modify the last fully-connected layer to output a single confidence score using the sigmoid function. The model applies binary cross entropy as its loss function.

We compute the hand-crafted features on an RGB image with size 224 × 224. We compute the color histogram separately on 4 non-overlapping spatial regions of the image using the hue, saturation, value (HSV) color space. The histogram for the lightness (i.e., value) channel has 8 evenly-spaced bins while the histograms for the other two each have 3 evenly-spaced bins. Concatenating all the histograms yields a final color feature with 288 dimensions. We also extract HOG [60] and LBP [61] descriptors for comparison experiment. To classify the action “dry hands” from the post-hygiene period using these hand-crafted features, we train a Random Forest.

**Testing:** We perform frame-level prediction to compare both the deep-learning model and hand-crafted feature Random Forest classifiers for the two ground-truth actions labeled either “dry hands” or “non-hygiene”. We evaluate the methods using precision, recall, and accuracy at the frame level by considering “dry hands” as a positive sample and “non-hygiene” as a negative sample.

From the results in Table 6, we observe that the CNN model performs best among all methods, even though the recall is a few percent lower than the other methods. As discussed in Section 3.3, the goal of detecting the “dry hands” action is to verify that it took place. If a non-hygiene action is mistakenly classified as drying hands, the system will fail to correctly assess the entire hand-hygiene process. Therefore, recall is less important than precision. To answer the Question 5 in the beginning Section 5, we conclude that the low-complexity CNN model is the preferred model to recognize “dry hands” action in the post-hygiene period.

## 6. Performance of the Two-Stage Hand-Hygiene System

In the previous Section 5, we explore the individual designs for each of the coarse and fine classifiers. The overall two-stage system for recognizing and evaluating hand-hygiene activities within untrimmed video appears in Section 4. In this section, we evaluate the performance of the entire two-stage system for detecting actions within untrimmed hand-hygiene videos. We first explain the experimental protocol, then report our experimental results for the overall system performance.

### 6.1. Experimental Protocol

In the first stage of the system, the wall coarse classifier uses the CNN model with RGB and MHI as inputs, and the chest coarse classifier applies a CNN model for binary classification on the “dry hands” action. In the second system stage, both the CNN model for wall and chest camera view are applied with VGG11 network architecture.

To create a point of comparison, we consider a baseline system that applies the second-stage VGG11 networks to the entire chest camera video and wall camera video. Each classifier densely processes the entire untrimmed video using non-overlapped 30-frame units. The classifier for the wall camera is only responsible to detect the action “touch faucet with elbow” action, and the classifier for the chest camera is applied to detect all other actions.

Recall that our goal for the two-stage system was to achieve similar performance to the baseline system, but with less computation. The baseline system applies VGG11 on every 30 consecutive frame units throughout the entire video, while the two-stage system applies VGG11 only when a simpler classifier would be insufficient. Therefore, we expect that both systems will achieve very similar detection accuracy. If so, it demonstrates that our coarse classifier successfully localizes the crucial hand-hygiene temporal parts, reducing the overall system complexity without sacrificing performance.

The overall performance of the two systems is evaluated on the first trial defined in Section 5.1.1.

### 6.2. Results and Discussion

We evaluate the overall performance of this system in two parts. In the first part, we evaluate only the two actions that require an estimate of how long they last; these actions are “rub hands with water” and “rub hands with no water”. We measure their detection performance using the Jaccard index, applied for units of 30 consecutive frames. In addition, we measure the average prediction error by computing the absolute difference between the detected time duration and ground-truth duration, averaged across all test videos. In the second part of evaluation, we consider the remaining actions for which we only need to confirm whether or not they happened. We evaluate these simply using the accuracy across all test videos, computed by dividing the number of correct predictions divided by the total number of predictions.

Table 7 and Table 8 show the evaluation of the rubbing actions. As we can observe, both the baseline and proposed two-stage system have similar performance in terms of both the Jaccard index and error in the estimated duration. This indicates that the first stage of the two-stage system could successfully localize the hand-hygiene period within the untrimmed video; the estimates of how long each action happens is consistent. Moreover, the ground-truth statistics for these two actions across the entire data set is shown in Table 9. Given the large average duration and standard deviation of these two actions, an estimation error of around 2 s is an reasonable result. To obtain further improvement, increasing the complexity of the CNN model and optimizing the hyperparameters might reduce the average error.

Table 10 demonstrates the accuracy across all test videos for the discrete actions of “apply soap”, “dry hands”, and “touch faucet with elbow”. These three actions have nearly identical performance for both the two-stage system and the baseline system. Therefore, no performance is lost by applying the low-complexity model for temporal localization.

Overall, the two-stage system and the baseline system achieve similar performance for recognizing each action and for estimating the duration of rubbing. However, with the support of the low-complexity CNN models for localization, the two refinement classifiers in our two-stage system only process 67.8% of the frames in the untrimmed videos. This is in contrast to the baseline that must densely process 100% of the frames in the untrimmed video, regardless of the duration of the hand-hygiene activity. Further, it should be noted that the videos we collected in this project were specifically designed to analyze hand hygiene, and as such they contain very little time spent on non-hygiene actions. Specifically, the average non-hygiene actions occupy only 28.1% of the total video duration, with the remaining 71.9% containing hand-hygiene activities. In more typical situations, where the hand hygiene would take less time relative to the overall collection of activities, the computational savings achieved by the temporal localization in our two-stage system would increase dramatically.

## 7. Conclusions

In this paper, we introduce the task of hand-hygiene action recognition from untrimmed video. We approach this problem by defining different levels of the task and design a system that performs hand-hygiene recognition at the standard level. To explore the efficiency of using different camera views on recognizing 7 hand-hygiene actions, we collected a dataset using three cameras and 100 participants. Using this dataset we are able to explore different deep-learning models on our hand-hygiene dataset with both egocentric and third person camera views. The results indicate both these camera views have their own unique advantages for recognizing certain action types. Thus, it is important to use both camera views for hand-hygiene action recognition.

Moreover, we also explore the realistic scenario in which we recognize hand-hygiene actions inside untrimmed video. We design a two-stage system to localize the target hand-hygiene regions and we apply deep-learning models from two camera views for the final recognition. In the first stage, a low-complexity CNN model is applied on the third-person view to segment the untrimmed video into three temporal periods. In the second stage, we assign these temporal periods to more complex CNN models trained for different camera views, so that each model only has to recognize the actions suited for that camera view. In the final evaluation, our two-stage system achieves similar performance to the baseline, which applies CNN models to densely process every second in the entire untrimmed video. We demonstrate that the two-stage system can efficiently filter out non-hygiene regions so that it only needs to apply complex CNN models to the crucial hand-hygiene temporal regions.

In the future, we plan to extend our current hand-hygiene system to different environments. The work presented in this paper focuses on system for hand-hygiene in a small enclosed restroom. However, other realistic hand-hygiene scenarios may also happen in industrial kitchens, restaurants, or laboratories. It is worth exploring how to refine our system to extract features that are robust against different illumination and background scenarios. This would allow the system to be easily applied to a new target environment to detect hand-hygiene actions without significant modifications. Furthermore, our current hand-hygiene system can also be extended to be applied on other aspects of food handling, like washing vegetables, by exploring the interaction between hands and objects. Moreover, the hand-hygiene system could also be expanded to consider the detail-level hand-hygiene task, as we described in Section 3.2.1, by taking advantage of more supportive information, such as depth and the location of human skeleton joints. 

## Figures and Tables

**Table 1:** Classifier accuracy for all three camera views; seven actions.

	**Camera**	Wall RGB	Chest RGB	Nose RGB	Number of Clips
**Action**					
Faucet elbow		94.03%	85.07%	83.58%	67
Faucet hand		91.76%	94.59%	93.41%	425
Rub water		93.73%	92.68%	91.38%	383
Rub nowater		87.21%	94.06%	86.30%	219
Soap		93.19%	88.48%	96.34%	191
Dry hand		54.76%	90.48%	76.19%	210
Non-hygiene		91.36%	93.39%	92.03%	590
Average		88.01%	92.57%	90.12%	NaN

**Table 2:** Classifier accuracy for models of different complexities; seven actions.

	Detection	Accuracy
Model		
Chest ResNet152		93.44%
Chest VGG19		93.68%
Chest VGG16		92.74%
Chest VGG11		94.38%
Wall ResNet152		86.65%
Wall VGG19		88.06%
Wall VGG16		88.52%
Wall VGG11		87.12%

**Table 3:** Classifier accuracy for the chest camera with spatial and temporal models; seven actions.

	Camera	Chest RGB	Chest Flow
Action			
Faucet elbow		85.07%	83.58%
Faucet hand		94.59%	89.41%
Rub water		92.68%	92.43%
Rub nowater		94.06%	94.98%
Soap		88.48%	76.96%
Dry hand		90.48%	94.28%
Non-hygiene		93.39%	95.59%

**Table 4:** Coarse wall classifier: Performance of RGB and RGB + MHI structures. **conv:** convolutional layer. **dw conv:** depthwise separable convolution. **fc:** fully connected layer.

	Evaluation	Frame-Level	Unit-Level
Structure			
RGB, 5 groups conv, 1 fc		89.96%	90.41%
RGB, 4 groups conv, 2 fc		86.45%	87.13%
RGB, 4 groups dw conv, 2 fc		83.50%	84.35%
RGB + MHI, 5 groups conv, 1 fc		91.21%	92.42%

**Table 5:** Coarse wall classifier: Performance of RGB and RGB + MHI between 3 actions at unit-level, both RGB and RGB + MHI use 5 groups of convolutional layers and 1 fc layer setting.

	Structure	RGB	RGB + MHI
Action			
faucet elbow		75.00%	85.71%
hand-hygiene		89.52%	93.08%
non-hygiene		89.17%	87.64%

**Table 6:** Coarse chest classifier: Prediction result for the “dry hand” action. Positive class: dry hand, negative class: non-hygiene.

	Evaluation	Precision	Recall	Accuracy
Model				
32 × 32 CNN		79.54%	61.31%	81.63%
Color histogram		54.32%	64.33%	70.49%
HOG		49.53%	65.38%	66.65%
LBP		50.51%	66.99%	67.58%

**Table 7:** Two-stage system: Average Jaccard index.

	Model	Baseline	Two-Stage System
Action			
Rub without water		0.8036	0.8036
Rub with water		0.8299	0.8307

**Table 8:** Two-stage system: Average mis-prediction in seconds.

	Model	Baseline	Two-Stage System
Action			
Rub without water		2.39	2.39
Rub with water		2.23	2.20

**Table 9:** Statistics for the duration of each action in the ground truth.

Action	Mean (secs)	Std. Dev. (secs)
Rub without water	16.40	11.83
Rub with water	13.72	7.93

**Table 10:** Two-stage system: Average detection accuracy.

	Model	Baseline	Two-Stage System
Action			
Soap		86.36%	86.36%
Dry hands		97.73%	93.18%
Faucet elbow		95.45%	95.45%
